# Studying the effect of lockdown using epidemiological modelling of COVID-19 and a quantum computational approach using the Ising spin interaction

**DOI:** 10.1038/s41598-020-78652-0

**Published:** 2020-12-10

**Authors:** Anshuman Padhi, Sudev Pradhan, Pragna Paramita Sahoo, Kalyani Suresh, Bikash K. Behera, Prasanta K. Panigrahi

**Affiliations:** 1 School of Physical Sciences, National Institute of Science Education and Research, HBNI, Jatni, Odisha 752050 India; 2 Physics Department, Indian Institute of Science Education and Research, Berhampur, Odisha 760010 India; 3 Maths Department, Indian Institute of Science Education and Research, Berhampur, Odisha 760010 India; 4 Department of Physics and Earth sciences, Universität Leipzig, Linnéstraße 5, Leipzig, 04103 Germany; 5 Bikash’s Quantum (OPC) Pvt. Ltd., Balindi, Mohanpur, Nadia, West Bengal 741246 India; 6 Department of Physical Sciences, Indian Institute of Science Education and Research Kolkata, Mohanpur, West Bengal 741246 India

**Keywords:** Mathematics and computing, Physics

## Abstract

COVID-19 is a respiratory tract infection that can range from being mild to fatal. In India, the countrywide lockdown has been imposed since 24th march 2020, and has got multiple extensions with different guidelines for each phase. Among various models of epidemiology, we use the SIR(D) model to analyze the extent to which this multi-phased lockdown has been active in ‘flattening the curve’ and lower the threat. Analyzing the effect of lockdown on the infection may provide a better insight into the evolution of epidemic while implementing the quarantine procedures as well as improving the healthcare facilities. For accurate modelling, incorporating various parameters along with sophisticated computational facilities are required. Parallel to SIRD modelling, we tend to compare it with the Ising model and derive a quantum circuit that incorporates the rate of infection and rate of recovery, etc as its parameters. The probabilistic plots obtained from the circuit qualitatively resemble the shape of the curve for the spread of Coronavirus. We also demonstrate how the curve flattens when the lockdown is imposed. This kind of quantum computational approach can be useful in reducing space and time complexities of a huge amount of information related to the epidemic.

## Introduction

COVID-19 (Coronavirus Disease 2019) is a disease caused by the virus strain known as SARS-CoV-2 (Severe Acute Respiratory Syndrome Coronavirus II). It has widespread implications on the human body in the form of respiratory issues, septic shock, co-morbidity arising from multiple-organ failure, and even death^1^. Routing back to its emergence in mainland China around the end of 2019, till May 2020, it has spread to over 210 countries resulting in a total of around 40 lakh cases with almost 3 lakh deaths due to the same. The World Health Organisation (WHO) declared it as a global pandemic on 11th March 2020, observing the rate at which it transmits. Various countries, including India, put forward extensive measures to curb the viral epidemic, by extensive tracing, testing and isolating the suspected ones while improving healthcare systems and imposing lockdowns. Government of India declared the countrywide lockdown on 24th March 2020 to reduce the virus’ rate of transmission. To tackle this global pandemic, the extent of spread and the time taken by the epidemic to reach its peak and other details must be well predicted so that the state can plan accordingly and fight against it.

Mathematical modelling can come handy in such processes, since they can provide one of the models as to how the epidemic ‘might’ evolve while analyzing the current set of available data. Any such attempt at prediction might be mathematically rigorous but still they come with a fairly high uncertainty as the assumptions and considerations in that model might not address all the complexities of the process. Thus a model and its application still have gaps to be covered^2,3^. Precise consideration of networks within a population while the model is being formulated nears an accurate prediction. Later, the prediction can be informed to the healthcare sector and the stakeholders for necessary implementations.

Here, we use the time-dependent SIRD (Susceptible–Infected–Recovered–Deceased) model to predict the evolution of this epidemic in India. The SIRD model is one among several compartmental studies in epidemiology^4^ such as SEIR, where E stands for exposed and rest staying the same; SEIRD, SIR, SIS model, etc. They have their origin from the Kermack–McKendrick theory of infection spread, a very rigorous statistical analysis performed in 1927. Here, the population is divided into various compartments, and their interactions are studied further. It is a simple yet an instrumental model of epidemiology since it takes into account various factors such as the rate at which the infection spreads, the rate at which the active cases recover, etc. Since more compartmentalization allows us to study the population in a detailed manner, models like SEIRD are always better than the chosen SIRD. However, SEIRD model needs the data for ‘Exposed’ individuals. Here, we use the crowd-sourced COVID 19 tracker^5^ database that provides the data for the number of infected (I), recovered (R) and deceased (D) individuals, while not being explicit about the number of exposed individuals. Even though the database gives the data for number of testings performed, it wasn’t found suitable enough to be considered as the tallies for exposed individuals since mild or asymptomatic carriers aren’t usually tested until found via contact tracing or travel history. Hence, SIRD model is suitable enough to be followed owing to its ease of data availability. In this current study, we use classical computation to demonstrate how a change in spread rates might stabilize figures related to infection, recovery, and death.

Several studies have been done with the available set of data of COVID-19 spread in India and other countries. Some of the notable works have used several other statistical approaches while some have used compartmental studies to model and predict the viral spread^6–14^.

Apart from its classic use in giving a forecast of the epidemic, in this study we use the infection, recovery and death data obtained for India to analyse how the growth pattern of the virus has changed over time and how these imposed constraints in the form of lockdown have lowered the viral transmission. We break the time scale into smaller intervals (a period of 10 days) and analyze them individually by applying a SIRD model over them, we find the values of the associated parameters in these periods, and thus obtain an approximate trend followed by the infection rates. We later use these curves to extrapolate and get a rough prediction of the infection rates for the next few days after the extended lockdown ends (17th May). Further, this set of predicted parameters are used to deduce the progress of the system in the near future with a fixed population. These deductions with current constraints on the system are put up against a system with no such constraints to demonstrate the efficacy of multi-phased lockdown on widening and delaying the peak of infection reporting.

Use of phenomena associated with quantum mechanics like superposition and entanglement for computational purposes is called quantum computation. Quantum circuit models aim to perform the same by using quantum bits (qubits) which unlike binary storage units, allow superposition of 2 distinct states. Thus qubits facilitate greater computational advantage than classical computation as they contain greater amounts of information due to the superposition. The state of the qubits are made to interact with various operators in the form of various quantum logic gates, meant to carry out a specific operation on any given qubit state (input) and give output accordingly^15,16^.

Quantum computation can be useful in the assessment of epidemics in network systems since an accurate prediction of a viral spread needs to encompass various factors that might pose complexity challenges in classical computation. Factors like quarantine measures, social distancing, population networking, self protection actions, etc, can give rise to a complex set of problems, challenging for a classical computer to solve. Assuming such factors could be easily fitted into quantum computation facilities, given its intrinsic ability to hold substantial information and parallelly process them, underlies this project’s hypothesis.

Attempts have been made to use quantum computational approach to model epidemics by Britt in 2010^17^. Here, he tried to model the viral network diffusion using the Non-linear Dynamical System (NLDs) while attempting to precisely control the person (represented by ‘nodes’ in the work) to person viral infection. Using all these, he built a quantum circuit, where he assigned 1 qubit to each of the nodes and could accurately simulate a sample population size with a given initial condition.

Parallel to the SIRD modelling in the study, we form an analogy to the Ising model of magnetic lattice to form a Hamiltonian. Then we build a quantum circuit to demonstrate how they are efficient enough to qualitatively show the nature of the epidemic through the obtained graphs as outputs. We also demonstrate how the considered parameters must be varied to reduce the number of infections when at its peak and also to delay the time by which viral spread peaks in the country. This delay of the peak with lowering of its height is given a term called ‘flattening the curve’ and this becomes crucial, as the affected population is more spread out for a given time interval. Hence, this might not flood the country’s healthcare facilities, unlike otherwise. The reduction of the number of cases at its peak also ensures that the current healthcare facility doesn’t face a shortage of resources while treating the patients.

To build the quantum circuit, we have used the IBM quantum experience platform. Various prototypes of quantum operators have been designed and have been made available through IBM quantum experience, a free-web based platform. Researchers have used it to their strength to experiment with circuits and also to simulate results which have furthered their research^18–33^.

We organize this paper as follows. The “theoretical background” section discusses the theoretical analysis of the SIRD compartmental model and the quantum computational approach. The “Implementation and results” section proposes how both of them have been implemented and what results were obtained from them. The “Interpretation and drawbacks” section is dedicated to a general discussion on the obtained results, including the drawbacks. Furthermore, at last, we conclude this article by citing the future implications of the proposal in “Conclusion” section. For additional references, Online Appendices A and B have been attached at the end.

## Theoretical background

### SIRD model

As already mentioned, the SIRD model is one of the compartmental models used in epidemiology. It divides the whole population into categories where S stands for the part of the population which is susceptible to being infected by the virus. I is the population that has been infected and has the potential to spread the infection. R is the group which has successfully recovered from the disease. D stands for the portion which has been deceased after getting infected due to it. N, the country’s total population assumed to be time-independent, is the sum of the susceptible, infected, recovered and the deceased.

To develop the operating mathematical model equations, some assumptions have been made to keep the model computationally simple. They are:

- The average birth rate and mortality rate of India have not been considered.
- The mode of transmission has been considered to be from person to person.
- Once a person has recovered from the disease, he/she has attained the immunity for infection, hence does not fall back to the category of susceptibles.

Here $\alpha$ is the rate at which the infection is transmitted to the susceptibles due to possible contact between infected and susceptible ones. $\beta$ indicates the rate at which the infected individuals recover, which is the reciprocal of the number of days in the treatment period. $\gamma$ describes the fatality of the virus as the rate at which infected individuals lose their life due to the viral infection. A study on the estimation of incubation period of Covid-19 virus by Lauer et al.^34^, has claimed that almost 97.5% of the cases show symptoms upon exposure by 11.5 days and by 14 days, 99% of the cases get detected. Hence in our article, we have assumed that the incubation period of the virus is 14 days for patients in India. From the chart presented above, we describe the SIRD modeling of the virus by the time rate of change of the different compartments of the population using coupled ordinary differential equations.

1 $$\begin{aligned} \frac{dS}{dt}= & {} -\frac{\alpha SI}{N}\nonumber \\ \frac{dI}{dt}= & {} \frac{\alpha SI}{N} -\beta I - \gamma I\nonumber \\ \frac{dR}{dt}= & {} \beta I\nonumber \\ \frac{dD}{dt}= & {} \gamma I \end{aligned}$$

2 $$\begin{aligned} N= & {} S+I+R+D \end{aligned}$$

The much talked about $R_{0}$ value of a viral epidemic is the total number of individuals to whom one infected person can transmit till he/she recovers. It can be calculated by using the expression $R_{0}$ = $\alpha$ x Incubation period (Incubation Period = $\frac{1}{\beta }$, if appropriate unit is used). If $R_{0}$ values become less than 1, then we *may* say that the situation will be under control and the disease now will eventually die down. It is so because the number of people infected per person would become less than the number of people recovering during that period.

We present the plot of the cumulative data of I, R, and D, obtain the parameters, $\alpha$, $\beta$, and $\gamma$ by the curves for fixed time intervals with multiple iterations. Later, we solve the mentioned differential equations for the multiple sets of parameters, in their corresponding time intervals, which nearly fits the data. Once the phase-wise plotting is done, we note down two sets of parameters, one corresponding to a no-lockdown/constraint system and another with some constraints. It must also be noted that this analysis assumes the occurrence of both extremities, and the period of the analysis is from fourth march 2020 to 12th May 2020.

### Ising model

On the other hand, we use the Ising model^35^ to build a quantum circuit to demonstrate the effect of ‘curve flattening’. The Ising model of atomic spin discusses the spin interaction of an individual atom in the lattice with its neighboring lattice points and how the spins behave in the presence of a magnetic field. Each can have an atomic spin of $+\frac{1}{2}$ or $-\frac{1}{2}$. The model describes the spin–spin coupling and the exchange interaction between the lattice points and the associated energy value to them. Interaction matrix depicts the fashion in which two lattice points interact with each other.

Here, Squillante et al.^36^, attempted to study the Covid-19 spread by comparing it with the Ising model, with the analogy of each atom as an individual. The spin of each lattice point^37^ (individual) describes whether the person is infected with the virus or not. A spin of $+\frac{1}{2}$ (p = probability of getting infected [success]) depicts that the person is infected with the virus and the spin of $-\frac{1}{2}$ (q= 1-p, probability of not being infected [failure]) indicates otherwise, while considering the total population being N. Hence, just like the Ising model, here also the spins (infected or susceptible individuals) interact with each other. The effect of magnetic field has not been considered here since it doesn’t carry any relevance in the analogy to the viral spread. It is so because of the closed nature of the population group in a country.

Since, in the case of viral epidemics, the infection is spread through ‘contact’ between individuals^38,39^, we assume a population of N, out of which ‘S’ are susceptible and ‘r’ people are infected. Here, we consider that when two infected people interact, the net interaction is 0 or no effect. So, a general probability distribution can be obtained from the Bernoulli’s equation as follows

3 $$\begin{aligned} P(X=r) = C^S_r*p^r*q^{S-r} \end{aligned}$$

From the previous SIRD model, we get a reproduction rate ($R_o= \frac{\alpha }{\beta }$) which also tells about the average number of secondary cases arising from primary cases in an entirely susceptible population, so we can consider $p=\frac{R_o}{S}$ or $pS=R_o$. The resultant equation turns out to be

4 $$\begin{aligned}&\lim _{S \rightarrow \infty } P(r)= \lim _{S \rightarrow \infty }\left[ \frac{R_o^r}{r!}\left( 1-\frac{R_o}{S}\right) ^S \left( 1-\frac{R_o}{S}\right) ^{-r}\frac{S!}{(S)^r(S-r)!}\right] \end{aligned}$$

Using Stirling approximation^40^ formula and further solving (4) we get a Poisson distribution (for further details, refer Online Appendix A),

5 $$\begin{aligned} P(r)= \frac{R_o^r e^{-R_o}}{r!} \end{aligned}$$

As analysed, new cases per day rise nearly exponentially to maximum value and then decrease, which shows the trend of a peak function, from which the equation could be seen as (5).

Coming back to Ising model of spin interaction, as we already discussed in brief, it talks about how spin systems interact with each other. Previously we also discussed our logic behind forming an analogy between a spin system and covid-19 interaction. Since they are analogous, we implement the general Hamiltonian for Ising model to the quantum circuit simulation of the epidemic. We can derive its time evolution factor with the probability of cases (or spin states) by implementing a Hamiltonian operator of its function dependent on time. As in the spin system, the spins interact with each other by the exchange interaction, it is defined by a Hamiltonian operator^41,42^ as

6 $$\begin{aligned} {\hat{H}}_r= & {} \sum _{n=1}^{N}A_{n}{(r)}{\hat{\sigma }}^x_{n}{\hat{\sigma }}^x_n+\sum _{n=1}^{N}B_n{(r)}{\hat{\sigma }}^y_n{\hat{\sigma }}^y_n + \sum _{n=1}^{N}C_n{(r)}{\hat{\sigma }}^z_n{\hat{\sigma }}^z_n \end{aligned}$$

where $A_n, B_n$ and $C_n$ are the strength of the exchange interactions (in the form of probability distribution) of the lattice points along the respective directions. Even though it is futile to consider different distributions of transmission along different directions when applied in Covid-19 simulation, still, we would first derive the quantum circuit for the generalized Hamiltonian of Ising model, and then later specialise it for Covid-19 scenario. Here, the interacting individuals are nothing but the lattice points with either one of the spins. The Pauli matrices determine the interaction of the spins (or individuals) with each other.

The strength of the exchange interaction decreases as the distance between these particles increases (which also helps in deducing that if people maintain distance, the spread of infection might decrease). In the neighboring region, we can almost assume that the interaction of one particle is almost similar to all of the neighbors, and the interaction is followed in all three directions, and thus the exchange interaction takes place; the stronger the exchange interaction more will be its infection rate.

Hence to find its correlation with the time evolution operator and deriving it into quantum circuits^43^ here, for n=1, we get a more straightforward form i.e.

7 $$\begin{aligned} {\hat{H}}_r=A_n{(r)}{\hat{\sigma }}^x_n{\hat{\sigma }}^x_n+B_n{(r)}{\hat{\sigma }}^y_n{\hat{\sigma }}^y_n+C_n{(r)}{\hat{\sigma }}^z_n{\hat{\sigma }}^z_n \end{aligned}$$

We apply the time evolution unitary operator on our Hamiltonian operator^44^

8 $$\begin{aligned} U(t)=e^{-\frac{iHt(2\pi )}{h}} \end{aligned}$$

by taking $\frac{h}{2\pi }=1$ and putting the Hamiltonian of (7) in unitary operator of (8) we deduce.

9 $$\begin{aligned} U(t)= & {} e^{-it(A_n{(r)}{\hat{\sigma }}^x_n{\hat{\sigma }}^x_n+B_n{(r)}{\hat{\sigma }}^y_n{\hat{\sigma }}^y_n+C_n{(r)}{\hat{\sigma }}^z_n{\hat{\sigma }}^z_n)} \end{aligned}$$

10 $$\begin{aligned}= & {} e^{-it(A_n{(r)}{\hat{\sigma }}^x_n{\hat{\sigma }}^x_n)}*e^{-it(B_n{(r)}{\hat{\sigma }}^y_n{\hat{\sigma }}^y_n)}*e^{-it(C_n{(r)}{\hat{\sigma }}^z_n{\hat{\sigma }}^z_n)} \end{aligned}$$

where $$\sigma ^x$$^45^= $$\begin{bmatrix} 0 &{} 1\\ 1 &{} 0 \end{bmatrix}$$, $$\sigma ^y$$= $$\begin{bmatrix} 0 &{} -i\\ i &{} 0 \end{bmatrix}$$ and $$\sigma ^z$$= $$\begin{bmatrix} 1 &{} 0\\ 0 &{} -1 \end{bmatrix}$$

Dividing the Eq. (10) into 3 parts as

11 $$\begin{aligned} U_1(t)= & {} e^{-it(A_n{(r)}{\hat{\sigma }}^x_n{\hat{\sigma }}^x_n)} \end{aligned}$$

12 $$\begin{aligned} U_2(t)= & {} e^{-it(B_n{(r)}{\hat{\sigma }}^y_n{\hat{\sigma }}^y_n)} \end{aligned}$$

13 $$\begin{aligned} U_3(t)= & {} e^{-it(C_n{(r)}{\hat{\sigma }}^z_n{\hat{\sigma }}^z_n)} \end{aligned}$$

On solving the Eq. (11)

14 $$\begin{aligned} U_1(t)= & {} e^{(-itA_n{(r)})({\hat{\sigma }}^x_n{\hat{\sigma }}^x_n)} \end{aligned}$$

which follows the condition of

15 $$\begin{aligned} e^{-i\theta A}=cos(\theta ) I -isin(\theta )A \end{aligned}$$

where I is the identity matrix and A is of same order of the identity matrix.

Here, A=$\sigma _x \otimes \sigma _x$, which follow the identity rule and has a order 4 and we break down it to

$$\begin{aligned} U_{1} (t) & = cos(A_{n} (r)t)I - isin(A_{n} (r)t)\sigma _{x} \otimes \sigma _{x} \\ & \left[ {\begin{array}{*{20}c} {cos(A_{n} (r)t)} &\quad 0 &\quad 0 &\quad { - isin(A_{n} (r)t)} \\ 0 &\quad {cos(A_{n} (r)t)} &\quad { - isin(A_{n} (r)t)} &\quad 0 \\ 0 &\quad { - isin(A_{n} (r)t)} &\quad {cos(A_{n} (r)t)} &\quad 0 \\ { - isin(A_{n} (r)t)} &\quad 0 &\quad 0 &\quad {cos(A_{n} (r)t)} \\ \end{array} } \right] \\ \end{aligned}$$

where U3^45^ gate controls The three parameters allowing the construction of any single-qubit gate, has a duration of one unit of gate time. In the Bloch sphere rotation, it can move through any plane controlled by (theta, gamma and phi) only once and its matrix form is represented by

$$\begin{aligned} \begin{bmatrix} cos\left( \frac{\theta }{2}\right) &{}\quad -e^{i\lambda }sin\left( \frac{\theta }{2}\right) \\ e^{i\phi }sin\left( \frac{\theta }{2}\right) &{}\quad e^{i(\phi +\lambda )}cos\left( \frac{\theta }{2}\right) \end{bmatrix} \end{aligned}$$

hence putting 2 CNOT gates i.e.

$$\begin{aligned} \begin{bmatrix} 1 &{}\quad 0 &{}\quad 0 &{}\quad 0\\ 0 &{}\quad 1 &{}\quad 0 &{}\quad 0\\ 0 &{}\quad 0 &{}\quad 0 &{}\quad 1\\ 0 &{}\quad 0 &{}\quad 1 &{}\quad 0 \end{bmatrix} \end{aligned}$$

and U3 with defined parameter in between, we can obtain the result of the above matrix of U1.

**Figure 1:**
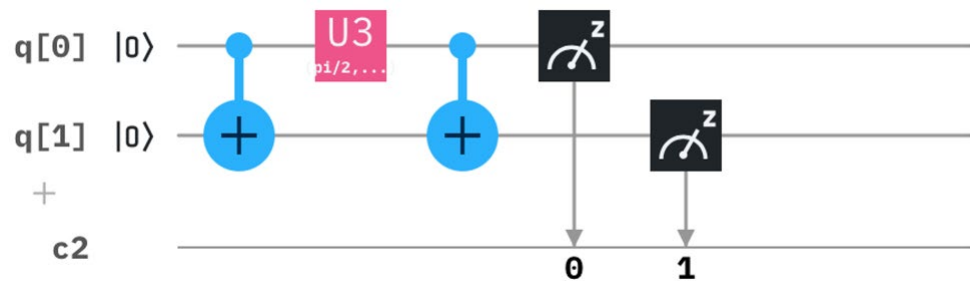
The derived circuit has a combination of a CNOT gate, U3 gate and a CNOT gate, where $\theta =2A_n(r)t$, $\phi =-\pi /2$ and $\lambda =\pi /2.$.

Hence, solving the matrix and comparing it with the U3 matrix we derive the following circuit^16^ as in Fig. 1.

Similarly, working for the Eq. (12) we get a reduced matrix format

$$\begin{aligned} U_{2} (t) & = cos(B_{n} (r)t)I - isin(B_{n} (r)t)\sigma _{y} \otimes \sigma _{y} \\ & \left[ {\begin{array}{*{20}c} {cos(B_{n} (r)t)} & 0 & 0 & { - isin(B_{n} (r)t)} \\ 0 & {cos(B_{n} (r)t)} & { - isin(B_{n} (r)t)} & 0 \\ 0 & { - isin(B_{n} (r)t)} & {cos(B_{n} (r)t)} & 0 \\ { - isin(B_{n} (r)t)} & 0 & 0 & {cos(B_{n} (r)t)} \\ \end{array} } \right] \\ \end{aligned}$$

similarly on solving this matrix manually, we get a combination of control U3 and anti control U3 matrix, where control U3 is a 4 $\times$ 4 matrix (I00U3) i.e.

$$\begin{aligned} \begin{bmatrix} 1 &{}\quad 0 &{}\quad 0 &{}\quad 0 \\ 0 &{}\quad 1 &{}\quad 0 &{}\quad 0 \\ 0 &{}\quad 0 &{}\quad cos\left( \frac{\theta }{2}\right) &{}\quad -e^{i\lambda }sin\left( \frac{\theta }{2}\right) \\ 0 &{}\quad 0 &{}\quad e^{i\phi }sin\left( \frac{\theta }{2}\right) &{}\quad e^{i(\phi +\lambda )}cos\left( \frac{\theta }{2}\right) \end{bmatrix} \end{aligned}$$

and anti control U3 is also a $4 \times 4$ matrix (U3 00I) i.e.

$$\begin{aligned} \begin{bmatrix} cos\left( \frac{\theta }{2}\right) &{}\quad -e^{i\lambda }sin\left( \frac{\theta }{2}\right) &{}\quad 0 &{}\quad 0 \\ e^{i\phi }sin\left( \frac{\theta }{2}\right) &{}\quad e^{i(\phi +\lambda )}cos\left( \frac{\theta }{2}\right) &{}\quad 0 &{}\quad 0 \\ 0 &{}\quad 0 &{}\quad 1 &{}\quad 0\\ 0 &{}\quad 0 &{}\quad 0 &{}\quad 1 \end{bmatrix} \end{aligned}$$

and placing this combination of matrix within 2 CNOT matrix with specified value of (theta, gamma , phi) we get the above matrix.

**Figure 2:**
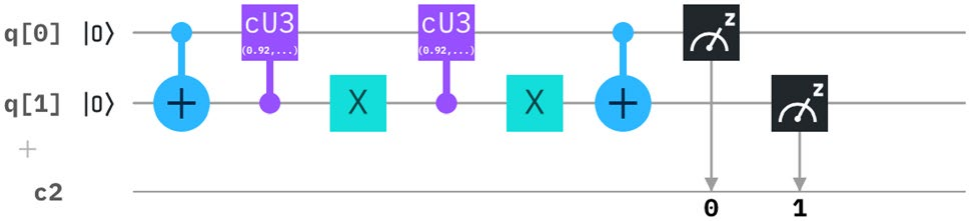
The derived circuit has a combination of a CNOT gate, control U3 gate, Anti-control U3 gate and a CNOT gate, where $\theta _1=2B_n(r)t$, $\phi _1=-\pi /2$ and $\lambda _1=\pi /2$ and $\theta _2=2B_n(r)t$, $\phi _2=\pi /2$ and $\lambda _2=-\pi /2$.

Where the equivalent matrix is reduced in the form of circuit^16^, as per Fig. 2.

Similarly, working for the Eq. (13), we get a reduced matrix format

$$\begin{aligned} U_{3} (t) & = cos(C_{n} (r)t)I - isin(C_{n} (r)t)\sigma _{z} \otimes \sigma _{z} \\ & \left[ {\begin{array}{*{20}c} {e^{{ - itC_{n} (r)}} } & 0 & 0 & 0 \\ 0 & {e^{{ - itC_{n} (r)}} } & 0 & 0 \\ 0 & 0 & 0 & 0 \\ 0 & 0 & 0 & {e^{{ - itC_{n} (r)}} } \\ \end{array} } \right] \\ \end{aligned}$$

Similarly, solving the matrix we get a very simplified form of U1 matrix, which is a $2 \times 2$ matrix which is shown as

$$\begin{aligned} \begin{bmatrix} e^{-i(-\frac{(\theta t)}{2})} &{}\quad 0\\ 0 &{}\quad e^{-i{\frac{(\theta t)}{2}}} \end{bmatrix} \end{aligned}$$

with a multiplicative factor at outside, and putting this U1^45^ between 2 CNOT gate ($4 \times 4$) on either side we get the above matrix.

**Figure 3:**
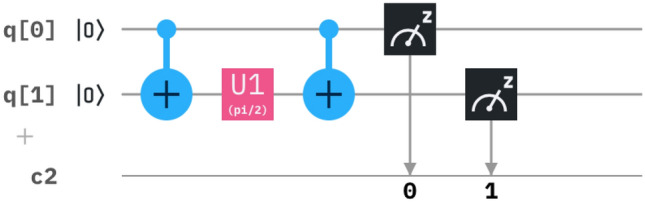
The derived circuit has a combination of a CNOT gate, U1 gate and a CNOT gate, where $\theta =2C_n(r)t$.

Where the equivalent matrix is reduced in the form of circuit as per Fig. 3.

**Figure 4:**
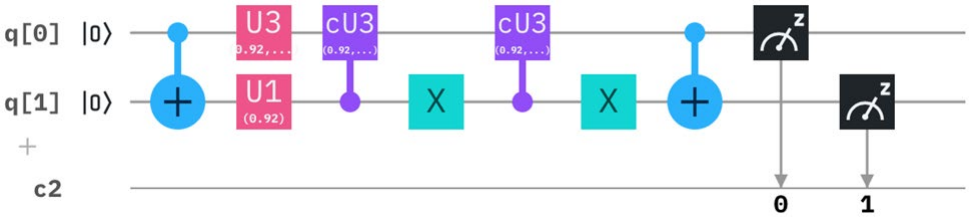
The equivalent circuit describes the Hamiltonian operator 7 in a N = 2 state.

And summing up all the circuits  we can add all the circuits together and as we know that two CNOT gates are equal to identity matrix so we can omit that and the resulting circuit somewhat looks like this as in Fig. 4.

**Figure 5:**
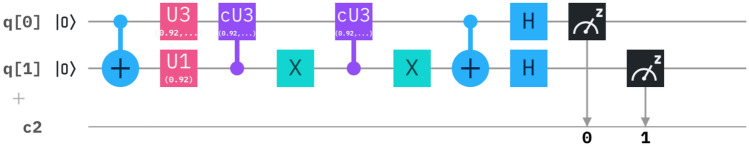
Measurement bases for the calculation of average values of ${<}\sigma _x\sigma _x{>}$ for N=2 in circuit.

Finding the average value^46,47^ helps find the energy eigenstate of the operator, which we can correspond to the number of infected people at a particular time. This energy eigenstate is sum total of all the average of $\sigma _x\sigma _x$, $\sigma _y\sigma _y$ and $\sigma _z\sigma _z$ with multiplication of the probability function (i.e. $A_n$, $B_n$ and $C_n$ respectively).

16 $$\begin{aligned} E_n= & {} A_n(r)t{<}\sigma _x\sigma _x{>}+B_n(r)t{<}\sigma _y\sigma _y{>} + C_n(r)t{<}\sigma _z\sigma _z{>} \end{aligned}$$

where, ${<}{>}$ = shows average value and ${<}\sigma _x\sigma _x{>}$= $P_{00} - P_{01} - P_{10} + P_{11}$ where $P_{00}$, $P_{01}$, $P_{10}$ , $P_{11}$ are the probability percentage of getting 00, 01,10 and 11 respectively, which is determined by putting 2 Hadamard gates at the end of the equivalent circuit, which is useful for moving information between the x and z bases, which is shown in following Fig. 5.

**Figure 6:**
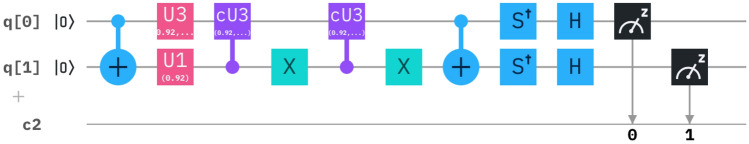
Measurement bases for the calculation of average values of ${<}\sigma _y\sigma _y{>}$ for N = 2 in circuit.

Similarly, ${<}\sigma _y\sigma _y{>}$ can be represented by putting a inverse S gate followed by a H gate in each qubit as it moves information from y to z bases, which is shown in Fig. 6.

**Figure 7:**
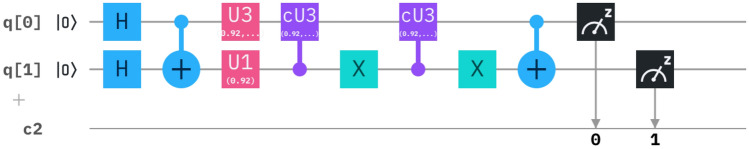
Measurement bases for the calculation of average values of ${<}\sigma _z\sigma _z{>}$ for N = 2 in circuit.

And for ${<}\sigma _z\sigma _z{>}$, we only measure the qubits in zz basis, which is shown in Fig. 7.

Now, to use them for simulation of viral transmission, as discussed earlier, we take a specific case of the generalized Hamiltonian where $A_n(r)$, $B_n(r)$ and $C_n(r)$, all three functions are considered to be P(r) which we have derived in (5). Hence, the Hamiltonian, whose time evolution we study for Covid-19 spread now turns-

17 $$\begin{aligned} {\hat{H}}_r=\frac{R_o^r e^{-R_o}}{r!}\big [{\hat{\sigma }}^x_n{\hat{\sigma }}^x_n+{\hat{\sigma }}^y_n{\hat{\sigma }}^y_n+{\hat{\sigma }}^z_n{\hat{\sigma }}^z_n\big ] \end{aligned}$$

## Implementation and results

### SIRD model

The first Covid-19 case was reported in India on 30th January 2020. Considering it as Day 1, on 4th March (the 33rd day), the number of cases jumped suddenly from 5 to 28. So, in this study, we start analyzing the data from March 4th onwards. We solve the SIRD equations using MATLAB. As Rajesh et al.^12^ pointed out in their SIRD model prediction of Covid-19 in India, that there is no reported error in the database, so we cannot use reduced chi-sq fitting for the above data. So, we employed the approach mentioned to carry out our analysis.

**Figure 8:**
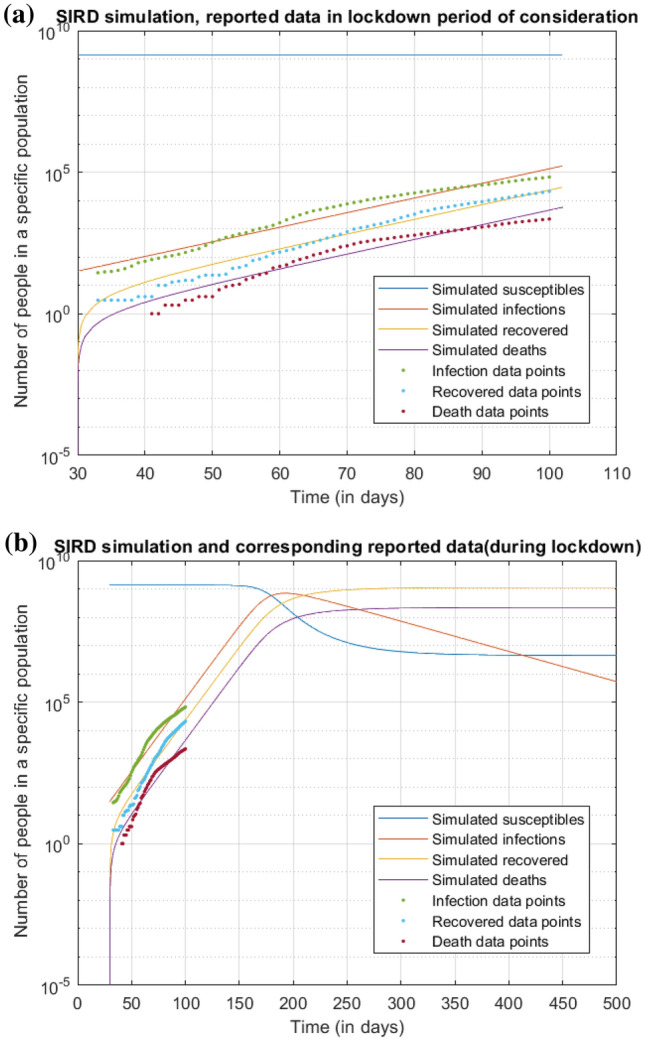
Simulating the prediction curves by using the best fit over the entire range of data. (**a**) depicts the data points and respective curves with $\alpha =0.144$, $\beta =0.021$ and $\gamma =0.0041$ (**b**) represents the same curve with the time axis extended upto 1000 days, thus giving a trend of the infection.

**Figure 9:**
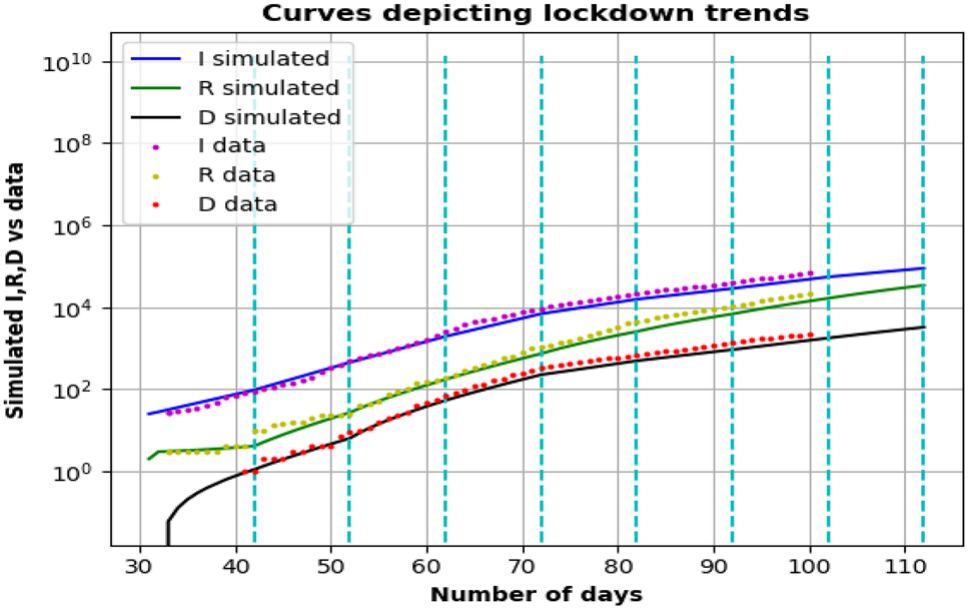
Simulating the prediction curves by using the best fit for every single interval obtained by dividing the time axis by the dashed cyan lines.

As per this fitting, in Fig. 8, the peak of the infection curve suggests that a maximum of $10^{9}$ people will get infected for a particular set of parameters. However, data plotting suggests a change in the concavity of the curve it follows, hence in its slope. Thus taking a single fitting for the whole period might not be a well scientific method. Hence, we tried to break the time interval into smaller legs (here we have chosen 10 days, because 10 day time period also would prove to be quite resistant to fluctuations in daily data and would help us get an overall trend) as per Table 1 and record the values of the parameters (Table 2) in the respective leg. The simulated curves are thus made to nearly fit the data points in Fig. 9.

**Table 1:** Distribution of lockdown into various legs.

Interval/Leg no.	Date	Day of spread
1	4 Mar–13 Mar	33–42
2	14 Mar–23 Mar	43–52
3	24 Mar–2 Apr	53–62
4	3 Apr–12 Apr	63–72
5	13 Apr–22 Apr	73–82
6	23 Apr–2 May	83–92
7	3 May–12 May	93–102

**Table 2:** Values of $\alpha$(t) and $\beta$(t) for various intervals for making the curve fit with the respective data sets.

Interval/Leg no.	$\alpha$	$\beta$
1	0.127	0.002
2	0.167	0.010
3	0.165	0.014
4	0.146	0.014
5	0.101	0.017
6	0.0825	0.020
7	0.091	0.024

By obtaining values of parameters, we proceed to make the curves for infection, recovery, and deaths that approximately match the data points available. Furthermore, we obtain $\alpha$ and $\beta$ as for each period i.e., $\alpha$(t) and $\beta$(t). Here, our only concern remains $\alpha$(t), and how it evolves in time as it is dependent on interactions between components of the population that’s being curbed and regulated during a lockdown^6^. $\beta$(t) and, on the other hand, is dependent on various other factors like the efficiency of treatment, the capacity of the healthcare system, and demographics of the considered population, which is not the point of discussion in this paper, hence not given much importance. Also, changes in their values are in the order of $10^{-3}$ if any were made during the fitting and duly presented.

As we already mentioned, we have assumed the incubation period of 14 days and that makes the $R_{0}$ value for the 3rd leg (March 24–April 2) 2.31, which falls to 1.274 by the end of the 7th leg. Hence, the lockdown has been able to contain the spread to some extent, but it should continue until it reaches a value closer to 1.

If we go on plotting the evolution of $\alpha (t)$ from the above data, we can see that a lockdown can successfully reduce the infection rate. To know the future of $\alpha$ under the lockdown, we consider the rate of infections of the several smaller periods under lockdown while excluding the first 2 data points for day 33–42 and 43–52 (since the lockdown nearly began on the day 53 of the arrival of the virus in India) and perform an exponential decay fit for the rest of the points. The reason of selecting an exponential decay function is the mathematical analysis performed for evolution of $\alpha$ over time (Refer Online Appendix B). The important conclusion we can draw from this plot, that the infection rate has been lowered by this lockdown. And since the points seem to obey the exponential decay fit upto some consistency, we can deduce that they confirm the mathematical analysis that the infection rate has the tendency to reduce exponentially when the population is under lockdown.

**Figure 10:**
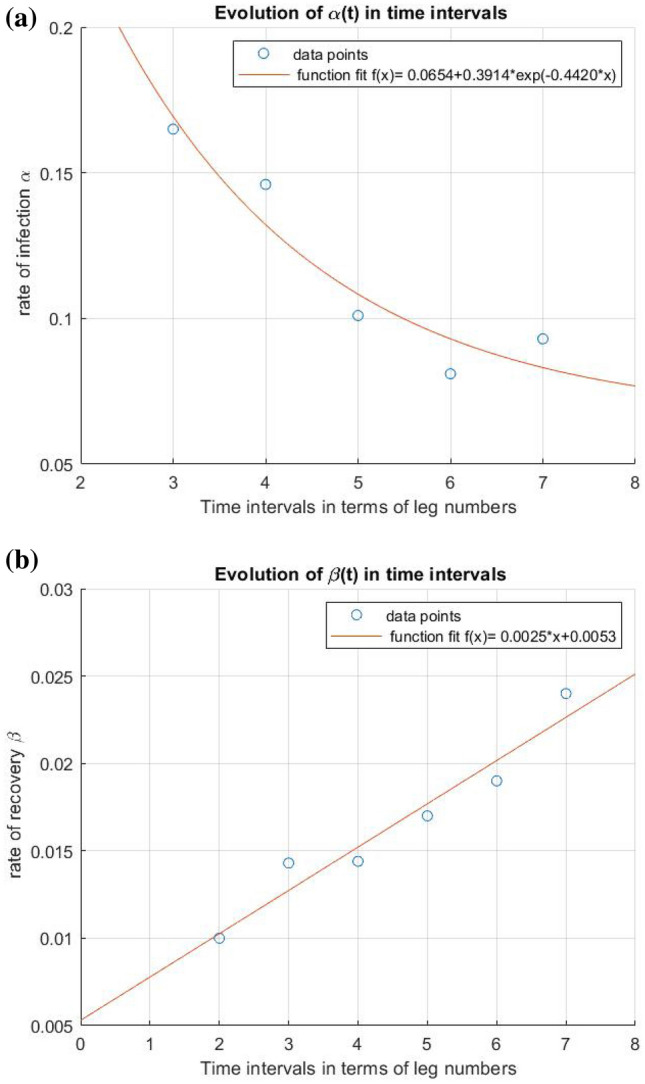
Curve fitting for studying the evolution of $\alpha$ and $\beta$ over time. (**a**) $\alpha$(t) being fit to an exponential decay function $y(x)=0.0654+0.3914\cdot e^{-0.4420x}$ (**b**) $\beta (t)$ being fit yo a straight line $y(x)=0.0025x +0.0053.$

As it can be seen from the plot, the points tend to have some deviation from the fit curve, which is due to the changing nature of the lockdown from time to time. Since we already know that 99% of the infected individuals start exhibiting symptoms in 14 days of getting infected, the true effect of any containment step can be visible only in the next 2 legs. We observe a pretty low drop for $\alpha$ in leg 4, while a heavy and significant drop can be noted for leg 5, which only portrays the effect of lockdown of its previous 2 legs. Gradual relaxation of the lockdown beginning from the 20th April (in the 6th leg) resulted in a mass movement of individuals and thus a higher infection rate could be noted for the 7th leg. The fit curve provides a very rough trend of the infection rate during the lockdown. The deviation of the point from the estimate curve can be attributed to the changing nature of the lockdown based on its variation in relaxation limit. We extrapolate this curve to have a general idea of what values alpha can presume in the coming legs. These values can help predict how the infection spreads over a long span of time. As shown in Fig. 10, we later use a straight-line fitting to extrapolate $\beta$ for subsequent legs. Here, we can interpret that the recovery rates have increased in a pretty uniform manner. It can be attributed to one or more aspects, such as improvement in the healthcare scenario, change in the demography of the infected individuals, or a boost in the quality and quantity of available treatment methods. Further studies and accurate models might be able to justify this trend (Fig. 10).

**Figure 11:**
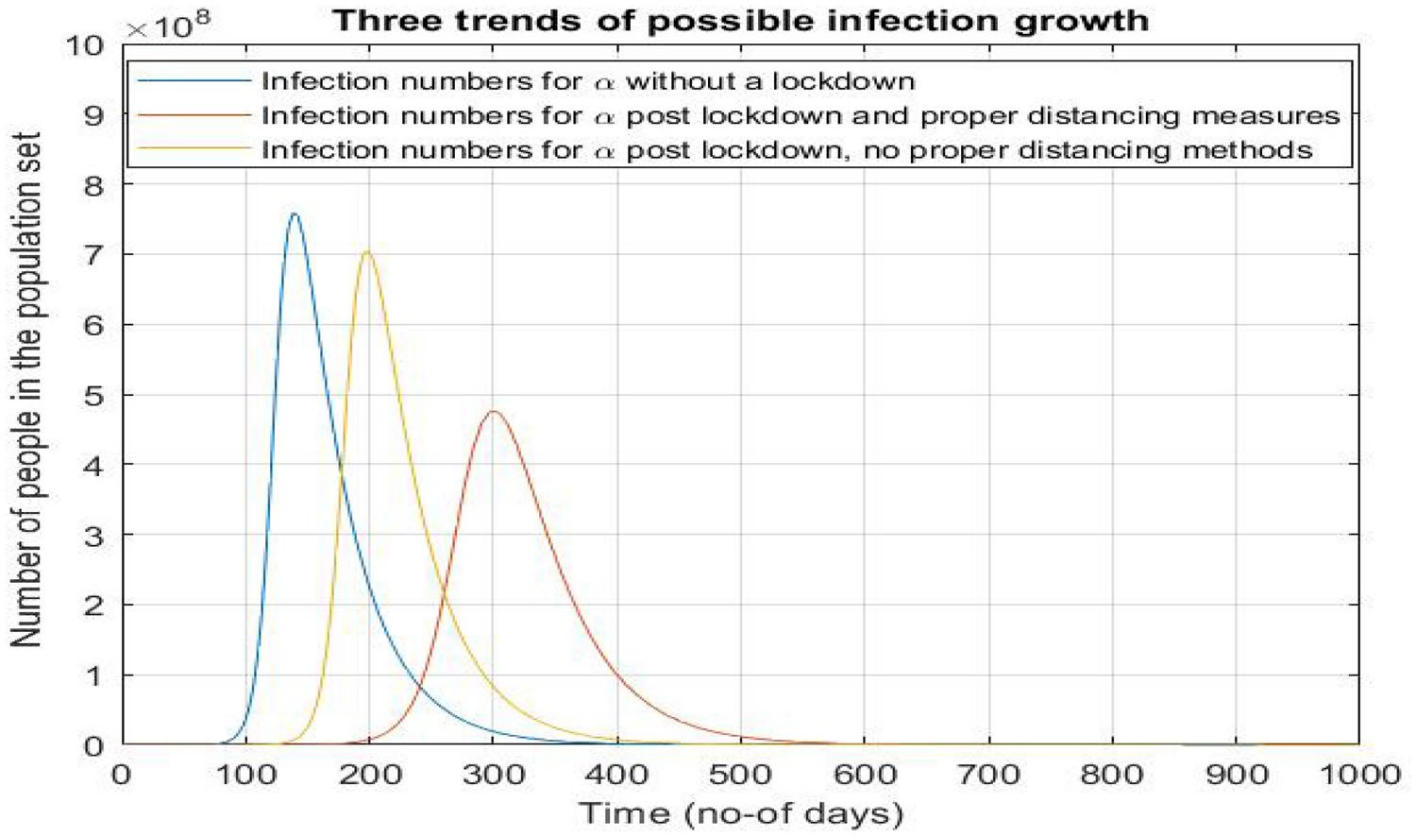
Analysis of number of infections upon various scenarios of lockdown. It can be observed that the number of cases at the peak almost halves with a distinct shift in the abscissa of peak, when the lockdown continues. When lockdown is removed all of a sudden (and assumed that the $\alpha = 0.165$, just like the value before lockdown), there is less decline in the number of cases at the peak with a subtle shift in the abscissa of peak.

We use the obtained values of $\alpha$ and $\beta$ for the next legs and simulate the SIRD curve and present it cumulatively. We have tried to analyze how the future numbers might be if the lockdown continues as it is until the disease is entirely null. After 3–4 legs further, the change in $\alpha$ would have started becoming negligible (and $R_{0}$ value slowly tending to 1) due to the asymptomatic nature of an exponential decay. The recovery rate also would have become nearly constant, since the healthcare facilities will achieve saturation in a few months. We have taken the values of the parameters at the end of the leg of period 132–142 to be constant throughout, for time to come. We extrapolated the curve to obtain a trend (Fig. 11).

Parallelly, we have studied the effect of the current lockdown being lifted. If the lockdown is lifted on May 17, we had assumed the other extreme that the infection rate might well reach the value it had before the imposition, but certainly not anything greater than that and plotted the curves.

### Ising model

**Figure 12:**
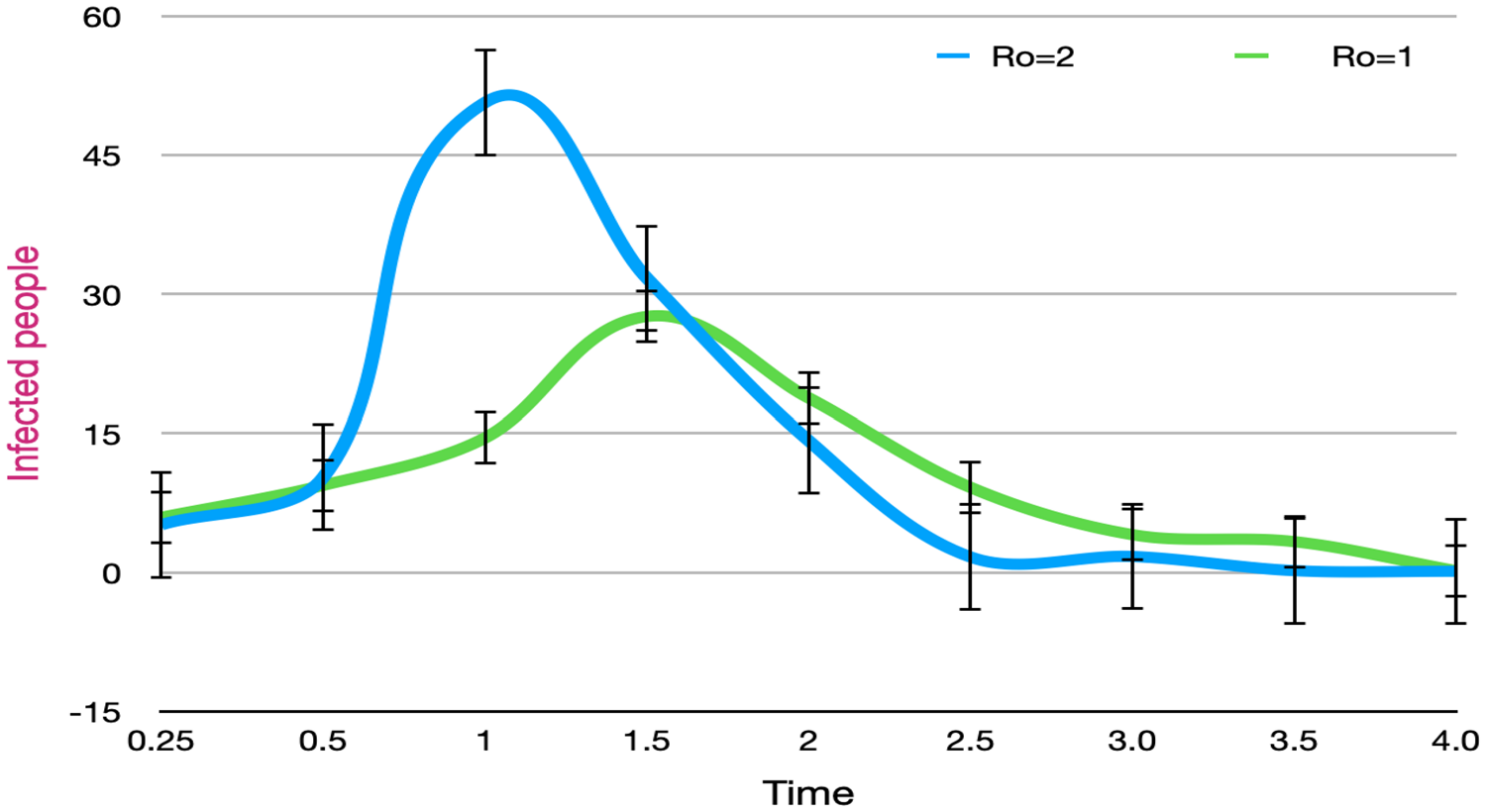
Used Hamiltonian being simulated using the IBM quantum experience platform, demonstrating the phenomena of curve flattening. A 2-D graph depicting the shift of the peak along X-axis when $R_{0}$ is decreased.

From Eq. (17) from the previous section, we have a Hamiltonian for the Covid-19 spread when compared to the Ising model. We discussed how the pandemic can be viewed as an Ising model simulation. From there on we assumed the Hamiltonian of the Infection spread being the same as that of Ising system without being under the influence of an external magnetic field. We went on to formulate the quantum circuit out of that Hamiltonian. In Eq. (6), we observed that the Hamiltonian is dependent upon the interaction functions (the probability distributions), so in order to simulate the infection we needed to find out the function representing the interaction among the atoms (or the people). But already prior to that, we used the binomial distribution analogy and derived a Poisson distribution function suitable enough for later being used in the Hamiltonian based quantum circuit to yield a curve (Fig. 12) quite with a considerable resemblance to the SIRD simulated curve and the infection curves for different countries. In the Hamiltonian, the parameter $R_{0}$ signifies the ratio of $\alpha$ to that of $\beta$. We can observe that when $R_{0}$ is lowered while keeping other variables and parameters constant, the peak of the curve shifts forward in the time axis with a reduction in its height and making it broader.

From these, we can infer that a decline in the rate of infection ($\alpha$) will infect lesser people than before, thus flattening the curve. Also, an increase in the rate of recovery ($\beta$) will imply an improvement in healthcare facilities, changing demographics of the infected patients, etc. and show a change in the height and position of the peak. Using proper parameters and constants, this can be a novel way to simulate approximate curves for a given situation. An analysis of epidemic spreads can be carried out with the inclusion of more and more factors that otherwise gets difficult for classical computers.

## Interpretation and drawbacks

The three graphs, with I, R, D show two trends post lockdown-one set of parameters without any distancing (constraints), and their respective carrier limits, and time is taken to attain them. The latter case depicts another set of parameters that have a few constraints imposed on them, as in the last phase of lockdown. So latter case parameters postponed the attainment of carrier limits and are not as rapidly growing as the former case. Also, the no lockdown case is started from the day of data collection, along with lockdown trends and post lockdown trends growing by two parameters to give an effective picture of what would have been the picture without a lockdown and how it would evolve once a lockdown is lifted off considering the extreme case scenario too. It is evident that the lockdown shouldn’t be lifted all of a sudden, as it will lead to a massive upsurge in the number of cases (nearly same as that of no-lockdown case) with a very narrow delaying of the peak.

The SIRD model discussed here has its own set of limitations pertaining to the assumptions made and the methods adopted while formulating it. The first limitation of this analysis is the use of the slope-estimation of data plotted curves to find the simulated curve in the lockdown period while obtaining a periodic split up of parameters. This might account for errors in the analysis. The assumptions like not considering birth and mortality rates, the permanent immunization of a recovered patient, unavoidable migration and interactions, etc. make the model lose its precision. Here, the susceptible are considered to be the entire population except the infected, recovered, and deceased individuals, initially at a time t. No considerations have been made to distinguish the exposed individuals out of the susceptible and model them accordingly. This might be a significant drawback of the model. The trend in recovery and its rate also is incomprehensible due to the lack of data on the demographics of the recovered patients in terms of their age, sex, etc. In this model, the number of tests performed and their rate has not been considered, but they might play a significant role in reporting the cases, hence the $\alpha$(t). Our N (the total population) has been assumed constant, but immigration, new birth, and deaths might vary the N, thus making us compromise on its accuracy. More efficient models like the SEIRD (Susceptible– Exposed–Infected–Recovered–Deceased), and higher compartmental models can be implemented for better prediction and analysis.

The quantum circuit design only gives a qualitative curve of an epidemic and the infection spread. With the proper use of the parameters and some constants, this can be used to simulate the curves for respective regions. Adding various complexities, out of which some are discussed in the above, is a matter of time and resources.

The earlier works on use of quantum computation like the Brits circuit, even though it stands out as it incorporates Euclidean distance between the nodes to define individual interaction among individuals, in this process it also assigns one qubit to each node thus adding to heavy operational space for analysis of bulk population. It becomes less feasible to process the same for a larger population, as obtaining data for individual interactions gets tougher as the population increases. Hence our circuit can perform better with a large population data without requiring data on individual interactions.

Here we have employed the quantum simulation of a Hamiltonian, since a Hamiltonian can contain a great deal of information, simulating the Hamiltonian of a large system (population) is far more suitable as it lets us incorporate fewer qubits, thus allowing us not to assign a qubit per individual. Hence, this work has the potential to be efficient enough for the use in large population forecasts. It also provides greater flexibility than previous works to later incorporate other factors and parameters like the number of tests, the age group of infected individuals, government measures in curbing the epidemic, etc. to make the prediction even more accurate. Our method, rather than dealing with individuals, deals with the $R_{0}$ value prevalent in the sample size and gives a probability distribution function instead, and thus is compact, and operates with less space and time complexity. For the same number of individuals, as our circuit would be requiring fewer qubits, it would also show less decoherence and gate error than that of the previous works.

The quantum circuit design in this study only gives the qualitative shape of a ‘usual’ curve of an epidemic infection spread with the proper use of the parameters and some constants, this can be used to simulate the curves for respective regions. SIRD model solely depends on the coupled ODEs and has many approximations to be considered as such. Ising’s model gives an interpretation of epidemiology in terms of solid state physics, which when further explored can give us a closer look at analysing the spread of infection, even via classical computation. Ising models make it feasible for a quantum computational approach to be implemented for epidemiological research, and we are aware of the reduction of space and time complexity brought in by quantum computation. Though quantum computational approach might not seem as a worthy replacement for classical counterpart in the current scheme of things, Ising model analogy and simulation of Hamiltonian opens a new field of discussion where quantum computation is incorporated. Adding various complexities, out of which some are discussed in the above, is a matter of time. With better and smarter use of quantum gates, efficient circuits can be made to minimize the space and time complexities while adding more parameters for accurate modelling. Thus it serves as a great base for further advancements in this field.

## Conclusion

Throughout this article, we discussed how the Ising model can be compared to epidemics like Covid-19 and be used in quantum computational approach. By forming a quantum circuit and running a simulation, we get the curve for the infection which is pretty much similar to real-world data. This gives a major motivation for work in this field and with further advances, quantum computation can come handy for epidemiological research due to its minimal space and time complexity. On the other hand, despite their limitations, use of the classical SIRD model provides us with a better analysis on how effective the lockdown has been in curbing the infection. By observing the trend of the infection during the lockdown, we also were able to forecast various scenarios of lockdown after 17th May 2020. It shows us the forecast for the reduction in the number of infected cases if lockdown is continued further.

COVID-19 has turned out to be a global crisis, affecting all the countries. The current model study report hints at a frightening upsurge of the viral epidemic in the times to come. Measures like quarantine and lockdown have been successful enough to reduce their impact, yet a lot needs to be taken care of. However, the current prolonged lockdown has started worrying national as well as global economies, pushing them into a tremendous crisis; hence the lockdown cannot be sustained forever. But as per our analysis, the lifting of lockdown shouldn’t be all of a sudden, and be more gradual in the approach. We must start practicing the concept of social distancing and personal hygiene to keep the viral spread at bay. In desperate times like these, researchers of all fields must come together and contribute towards finding more information regarding the virus through experiments and data analysis. This shall let us be more aware and help us in tackling the risk. We sincerely hope that, just like previous global pandemics, we can pass through this with the advent of science and technology.

## Supplementary Information

Supplementary Information.

## Data Availability

The data that support the findings of this study are available from the authors (sudev18@iiserbpr.ac.in, bikash@bikashsquantum.com) upon reasonable request.
