# Supplementary material for: Studying the effect of lockdown using epidemiological modelling of COVID-19 and a quantum computational approach using the Ising spin interaction
Source: Sci Rep. 2020 Dec 10;10:21741. doi: 10.1038/s41598-020-78652-0 (PMC7729942; doi:10.1038/s41598-020-78652-0)
Supplement: Supplementary file 1 — Supplementary Information. [file 41598_2020_78652_MOESM1_ESM.pdf]

A Appendix (On finding the probability distribution function for implementation of Ising model)

p: The probability of getting infected.

q: The probability of not getting infected i.e. $q = 1 - p$

Hence, according to binomial distribution,

$P(X = r) = C_r^S * p^r * q^{S-r}$

$\implies P(X = r) = \frac{S!}{(S-r)!r!} \frac{R_0^r}{S^r} (1 - \frac{R_0}{S})^{S-r}$

Assuming the susceptible to be infinite, R_0 is a constant.

$$
\begin{aligned}
\lim_{S\to\infty} P(r) &= \lim_{S\to\infty} [\frac{R_o^r}{r!}(1 - \frac{R_o}{S})^S (1 - \frac{R_o}{S})^{-r} \frac{S!}{(S)^r (S-r)!}] \\
&= \frac{R_o^r}{r!} \lim_{S\to\infty} (1 - \frac{R_o}{S})^S (1 - \frac{R_o}{S})^{-r} \frac{S!}{(S)^r (S-r)!}] \\
&= \frac{(R_o)^r e^{-R_o}}{r!} \lim_{S\to\infty} \frac{S!}{(S)^r (S-r)!} \\
\left(As \lim_{S\to\infty} (1 - \frac{R_o}{S})^S = e^{-R_o} \right)
\end{aligned} \tag{18}
$$

Using: Stirling's-approximation-formula i.e. $S! = \sqrt{2\pi S} e^{-S} S^S$

Then,

$$\begin{aligned}
\implies P(r) &= \frac{(R_o)^r e^{-R_o}}{r!} \lim_{S\to\infty} \frac{\sqrt{2\pi S} e^{-S} S^S}{S^r \sqrt{2\pi(S-r)} e^{-S+r} (S-r)^{S-r}} \\
&= \frac{(R_o)^r e^{-R_o}}{r!} \lim_{S\to\infty} \frac{e^{-S+S+0.5} S^{S-r+0.5}}{(S-r)^{S-r+0.5}} \\
&= \frac{(R_o)^r e^{-R_o}}{r!} e^{-r} \lim_{S\to\infty} (\frac{S}{S-r})^{S-r+0.5} \\
&= \frac{(R_o)^r e^{-R_o}}{r!} e^{-r} \lim_{S\to\infty} \frac{1}{(1-\frac{r}{S})^S} \\
&= \frac{(R_o)^r e^{-R_o}}{r!} e^{-r} e^r \\
&= \frac{(R_o)^r e^{-R_o}}{r!}
\end{aligned}$$

B Appendix (Evolution of α w.r.t. time: An analytical approach)

Considering a process defined by the following parameters:

$r_1(t)$=probability that a susceptible person becomes infected upon primary contact.

$r_2(t)$=probability that the next interaction of the susceptible is with an infected person.

α(t)=overall probability that susceptible becomes infected.

N: total population under consideration

S: total number of susceptibles in the given population

In a homogeneous mixing of population , $r_2(t) = 1 - \frac{S}{N}$

A random susceptible will come in contact with 'n' individuals in a time interval Δt with a frequency λ.

So, the process is a poisson distribution with a rate of λΔt.

The number of infected persons, 'j' out of these 'n' will have a binomial distribution (n,r_2(t));

Finally,

$$\alpha \propto \sum_{n=0}^{\infty} \frac{e^{-\lambda \Delta t}(\lambda \Delta t)^n}{n!} \left(\sum_{j=0}^{\infty} {}^nC_j (r_2)^j (1-r_2)^{n-j} (r_1)^j \right) \tag{19}$$

Introducing $r_1 = 1 - r_1\prime$

$$\alpha \propto \sum_{n=0}^{\infty} \frac{e^{-\lambda \Delta t}(\lambda \Delta t)^n}{n!} \left(\sum_{j=0}^{\infty} {}^nC_j (r_2)^j (1-r_2)^{n-j} (1-r_1\prime)^j \right) \tag{20}$$

For $z_1 = r_1\prime(t)$, a z-transform would result in:

$$\alpha \propto \sum_{n=0}^{\infty} \frac{e^{-\lambda \Delta t}(\lambda \Delta t)^n}{n!} (1 - r_2 r_1\prime)^n \tag{21}$$

For $z_2 = 1 - r_2 r_1\prime$, a z-tranform would result in:

$$\alpha = e^{-\lambda \Delta t r_2 r_1\prime} \tag{22}$$

where $r_1\prime = 1 - r_1$

Since time intervals are considerably small,consider

$$\alpha = e^{-c \Delta t} \tag{23}$$

where $c = -\lambda r_2 r_1\prime$
